# OA13 Trust your gut: it has the answer

**DOI:** 10.1093/rap/rkae117.013

**Published:** 2024-11-05

**Authors:** Zoe Syrimi, Susannah Brockbank, Alastair Scott, Julie Dawson

**Affiliations:** Mersey and West Lancashire Teaching Hospitals NHS Trust, St Helens, United Kingdom; Mersey and West Lancashire Teaching Hospitals NHS Trust, St Helens, United Kingdom; Lancaster University, Lancaster, United Kingdom; Mersey and West Lancashire Teaching Hospitals NHS Trust, St Helens, United Kingdom; Mersey and West Lancashire Teaching Hospitals NHS Trust, St Helens, United Kingdom

## Abstract

**Introduction:**

We report the case of a 51-year-old man who presented over seven years to multiple specialities, including rheumatology on several occasions, with arthralgia, fevers and weight loss. He was found to have raised inflammatory markers, lymphadenopathy and pericardial and pleural effusions but despite extensive investigation over this time, the cause for his presentation remained elusive. After many years, he developed gastrointestinal symptoms, which led us to the unifying diagnosis of Whipple’s disease.

**Case description:**

This patient initially presented to rheumatology in 2017 with left ankle pain. His only medical history was renal calculi. He was diagnosed with gout and osteoarthritis and was discharged.

Two years later, he presented with fevers. Investigations revealed raised inflammatory markers, small pericardial and pleural effusions and small-volume upper abdominal lymphadenopathy. He was treated with colchicine for pericarditis with some improvement.

He presented again in 2021 with chest and flank pain and breathlessness. CTPA revealed an ongoing small pericardial effusion and small-volume intrathoracic and intra-abdominal lymphadenopathy. Inflammatory markers remained raised (CRP 30-50, ESR 20-30). He had now begun to lose weight. He was referred to the cancer of unknown primary service, who monitored his lymphadenopathy.

In January 2022, he underwent an open biopsy of a mesenteric lymph node, which revealed non-caseating granulomas and no evidence of malignancy. He was referred again to rheumatology due to ongoing migratory joint pains in his wrists, ankles and knees. Rheumatoid factor, anti-CCP, antinuclear antibody and serum ACE had been negative on several occasions. He underwent a PET scan that revealed persistent lymphadenopathy with low to moderate avidity, borderline splenomegaly and a slightly prominent marrow signal. He then had a bone marrow biopsy that showed no significant abnormality.

In December 2023, we settled on a probable diagnosis of sarcoidosis. He was treated with a trial of steroid without much symptomatic improvement and there was a plan to initiate Methotrexate. However, he then developed nausea and vomiting and was noted to have a new iron deficiency anaemia. He was referred to gastroenterology and underwent an upper endoscopy, which was macroscopically normal, but microscopically showed findings in keeping with Whipple’s disease (WD) with PAS-positive staining. Duodenal biopsy PCR confirmed this. He is now awaiting input from the infectious diseases team and long-term antibiotic treatment.

**Discussion:**

WD is caused by the bacterium *Tropheryma whipplei*. There are four commonly recognised manifestations of *T. whipplei* infections: 1) classic WD, 2) localised chronic infections, 3) acute infections and 4) asymptomatic carriage. Our patient has classic WD, and his case exemplifies the disease course and diagnostic challenges.

Classic WD is a rare, chronic, multisystem infectious disease. It is reported as most often affecting middle-aged white males of European ancestry. It can be divided into an early phase with intermittent arthralgias or arthritis and fever, a middle phase with gastrointestinal symptoms and weight loss, and a late phase with predominant neurological symptoms. Arthritis is the presenting symptom in 60-70% of cases and occurs in 90% of all cases. Given this, around half of patients are initially misdiagnosed as having an inflammatory rheumatic disease. As in the case of our patient, joint manifestations do not correlate with intestinal symptoms and can precede intestinal manifestations by a mean interval of six years. Joint involvement in WD is typically a migratory oligo- or polyarthritis or arthralgia primarily involving large and medium joints, most commonly the knees, ankles and wrists. Less commonly affected joints are elbows, hips and shoulders.

WD should be considered in all patients with the four cardinal symptoms of arthralgias, diarrhoea, abdominal pain, and weight loss after more common differentials have been excluded. In patients without gastrointestinal symptoms, having suspicion of the disease is more difficult; however, consideration should be made in patients with seronegative arthritis, who do not respond to immunosuppressive treatment. Other clinical syndromes in which WD should be considered include fever of unknown origin, chronic serositis, generalised lymphadenopathy, progressive central nervous system disease or early onset cognitive deficits.

Although histology revealing non-caseating granulomas is the hallmark of sarcoidosis, they are non-specific and can be found in many other diseases.

**Key learning points:**

• WD has a reputation as a great mimicker of many diseases and there is still often a considerable delay to diagnosis, even in typical cases, given the natural history of the disease and its rarity. WD should be considered in patients with seronegative arthritis not following a typical disease course, particularly if they develop gastrointestinal symptoms. WD is also a pertinent differential in patients with persistent constitutional upset with unexplained lymphadenopathy or serositis. It should be remembered that non-caseating granulomas can be found in many diseases and that sarcoidosis remains a diagnosis of exclusion among granulomatous disorders.

